# A pilot study of ^68^ Ga-PSMA-617 PET/CT imaging and ^177^Lu-EB-PSMA-617 radioligand therapy in patients with adenoid cystic carcinoma

**DOI:** 10.1186/s13550-022-00922-x

**Published:** 2022-08-19

**Authors:** Guochang Wang, Mengjiao Zhou, Jie Zang, Yuanyuan Jiang, Xiaohong Chen, Zhaohui Zhu, Xiaoyuan Chen

**Affiliations:** 1 Department of Nuclear Medicine, State Key Laboratory of Complex Severe and Rare Diseases, Beijing Key Laboratory of Molecular Targeted Diagnosis and Therapy in Nuclear Medicine, Peking Union Medical College Hospital, Chinese Academy of Medical Sciences, Peking Union Medical College, Beijing, 100730 China; 2 Department of Otolaryngology Head and Neck Surgery, Key Laboratory of Otolaryngology Head and Neck Surgery, Ministry of Education, Beijing Institute of Otolaryngology, Beijing Tongren Hospital, Capital Medical University, Beijing, 100730 China; 3 Departments of Diagnostic Radiology, Surgery, Chemical and Biomolecular Engineering, and Biomedical Engineering, Yong Loo Lin School of Medicine and Faculty of Engineering, National University of Singapore, Singapore, 119074 Singapore; 4 Clinical Imaging Research Centre, Centre for Translational Medicine, Yong Loo Lin School of Medicine, National University of Singapore, Singapore, 117599 Singapore; 5 Nanomedicine Translational Research Program, NUS Center for Nanomedicine, Yong Loo Lin School of Medicine, National University of Singapore, Singapore, 117597 Singapore

**Keywords:** ^68^ Ga-PSMA-617 PET/CT, ^18^F-FDG PET/CT, Adenoid cystic carcinoma, ^177^Lu-EB-PSMA-617

## Abstract

**Background:**

This pilot study was designed to evaluate the diagnostic value of ^68^ Ga-PSMA-617 and ^18^F-FDG PET/CT in adenoid cystic carcinoma (ACC) and to assess the safety and therapeutic response to PSMA radioligand therapy (RLT) in ACC patients.

**Methods:**

Thirty patients pathologically diagnosed with ACC were recruited into the cohort. Each patient underwent ^68^ Ga-PSMA-617 and ^18^F-FDG PET/CT within 1 week. The number and SUVmax of PET-positive lesions were recorded and compared. Four patients accepted RLT using ^177^Lu-EB-PSMA-617, in a dosage of approximately 1.85 GBq (50 mCi) per cycle for up to 3 cycles.

**Results:**

Compared with ^18^F-FDG, ^68^ Ga-PSMA-617 revealed more PET-positive extrapulmonary tumors (157 *vs.* 141, *P* = 0.016) and higher SUVmax (8.8 ± 3.6 *vs.* 6.4 ± 4.2, *P* = 0.027). However, ^68^ Ga-PSMA-617 revealed less PET-positive pulmonary lesions (202 *vs.* 301, *P* < 0.001) and lower SUVmax of tumors (3.1 ± 3.0 *vs.* 4.2 ± 3.9, *P* < 0.001) than ^18^F-FDG. The combination of ^68^ Ga-PSMA-617 and ^18^F-FDG can detect 469 PET-positive lesions, which was superior to each alone (469 *vs.* 359 *vs.* 442, *P* < 0.001). Two patients achieved remarkable response after PSMA RLT, while the other two patients showed reduced tumor uptake of recurrent foci, lung and liver metastases, whereas increased SUVmax of bone metastases.

**Conclusions:**

^68^ Ga-PSMA-617 PET/CT is a valuable imaging modality for the detection of ACC and combining with ^18^F-FDG PET/CT will achieve a higher detection efficiency. PSMA RLT may be a promising treatment for ACC and is worth of further investigation.

*Trial registration*: Diagnosis of Adenoid Cystic Carcinoma on ^68^ Ga-PSMA-617 PET-CT and Therapy With ^177^Lu-EB-PSMA-617 (NCT04801264, Registered 16 March 2021, retrospectively registered).

*URL of registry*: https://clinicaltrials.gov/ct2/show/NCT04801264.

## Background

Adenoid cystic carcinoma (ACC) is a rare type of epithelial tumor mostly originated from salivary glands, accounting for 1% of total head and neck cancers [1, 2]. Histologically, ACC comprises tubular, cribriform, and solid patterns, and it is generally recognized that a solid growth pattern indicates an advanced tumor grade and a worse prognosis [3, 4]. ACC exhibits the characteristics of slow growth, extensive invasion, frequently local relapse, and a relatively high probability of distant metastases [5]. At present, the main treatment for ACC is surgical resection, yet ACC tends to spread along nerve tracts, involving vital structures and organs in the surgical field, which presents challenges to achieve complete radical resection. Recurrent tumor requires re-surgery or local radiation therapy, which has become a clinical routine treatment. Even so, the rate of 5-year distant metastasis is as high as 52% [6–8]. Chemotherapy and targeted therapy are not effective against ACC so far. Therefore, once a patient is diagnosed with metastatic ACC, the prognosis is poor, with a median survival of 20–32 months [2, 9, 10]. Hence, early accurate diagnosis, staging, and effective adjuvant treatment are crucial to the management of ACC patients and improve the prognosis.

**Fig. 1:**
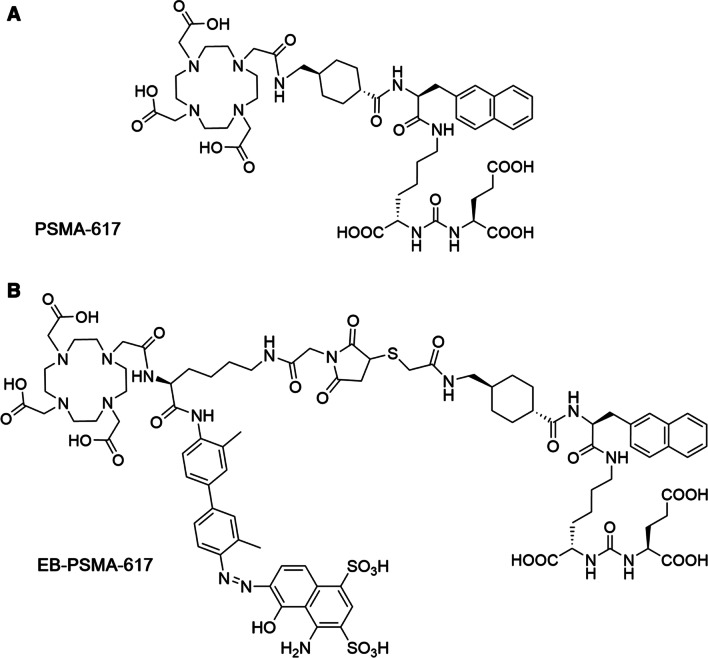
Structural formula of PSMA-617 (**A**) and EB-PSMA-617 (**B**)

In the past few decades, remarkable advances have been made in precision medicine based on positron emission tomography (PET) imaging, and the significance of ^18^F-fluorodeoxyglucose (^18^F-FDG) PET/computed tomography (CT) in the diagnosis and staging of various tumors is well recognized. However, not all ACC lesions exhibit identifiable FDG uptake [11, 12]. Prostate-specific membrane antigen (PSMA), also known as folate hydrolase I or glutamate carboxypeptidase II, is overexpressed by tumor cells or neovascular endothelial cells, such as prostate cancer (PCa), ACC, renal cell carcinoma, and hepatocellular carcinoma [13–19]. In most ACC lesions, PSMA expression is observed on cytomembrane of tumor cells rather than the vasculature [13, 20, 21]. Some previous studies of immunohistochemistry of primary, local recurrent, and distant metastatic ACC confirmed PSMA expression in these tumors [22, 23]. Van Boxtel et al*.* reported the percentage of PSMA-positive tumor cells for primary ACC and metastatic lesions was 7.5% (range 0–90%) and 5% (range 0–80%). Besides, tumor-associated neovasculature exhibited no PSMA expression [20]. Another research enrolled 9 patients revealed that PSMA expression was seen in all patients, mainly in cytoplasmic or concentrated at the luminal side of the cell membrane, varied widely between 5 and 90%, and a median of 30% of the primary tumor cells (IQR 15–70%) demonstrated PSMA expression [13]. Some studies have demonstrated that PSMA PET/CT is a valuable modality to detect and visualize ACC lesions and proposed the possibility of radioligand therapy (RLT) in ACC patients [13, 20]. Up to now, PSMA-targeted RLT against PCa has achieved encouraging beneficial effects [24–26]. One of the most widely studied PSMA radiopharmaceuticals is ^177^Lu-PSMA-617. As a diagnostic tracer, PSMA-617 is cleared quickly from the blood. Therefore, PSMA RLT based on ^177^Lu-PSMA-617 requires higher doses, which may cause obvious systemic toxicity. We modified PSMA-617 by conjugating a truncated Evans blue (EB) molecule and a DOTA chelator and then labeled it with ^177^Lu to synthesize ^177^Lu-EB-PSMA-617, the molecular structure of which is shown in Fig. 1. EB can bind to albumin to slow down its plasma clearance rate. Hence, EB-PSMA-617 could increase the tumor accumulation and reduce the total dosage of ^177^Lu, thereby precisely focusing as much radiation as possible on the tumor and improving the utilization rate of ^177^Lu. A previous study confirmed that the accumulated radioactivity of ^177^Lu-EB-PSMA-617 in tumor was about threefold higher than that of ^177^Lu-PSMA-617. However, the absorbed doses of ^177^Lu-EB-PSMA-617 in the red bone marrow and kidneys were also significantly higher than those of ^177^Lu-PSMA-617 [27]. Clinical studies have demonstrated the remarkable efficacy of ^177^Lu-EB-PSMA-617 in the treatment of PSMA-positive PCa [27, 28], which has led to the question whether ^177^Lu-EB-PSMA-617 could also achieve satisfactory therapeutic efficacy in ACC. It is essential to carry out a prospective trial of PSMA RLT in ACC patients.

This pilot study was designed to further evaluate the diagnostic performance of ^68^ Ga-PSMA-617 PET/CT in ACC in a head-to-head comparison with^18^F-FDG PET/CT and to preliminarily assess the safety of and therapeutic response to PSMA RLT in patients with ACC.

## Materials and methods

### Patients

This study was approved by the institutional review board of Peking Union Medical College Hospital, Chinese Academy of Medical Sciences and Peking Union Medical College (no. ZS-2532), and registered at clinicaltrials.gov (NCT04801264).

Patients with pathologically diagnosed ACC were prospectively recruited to undergo ^68^ Ga-PSMA-617 and ^18^F-FDG PET/CT. Written informed consent was obtained from each subject.

Regarding the inclusion criteria for PSMA RLT, ACC lesions with high PSMA uptake confirmed by ^68^ Ga-PSMA-617 PET/CT, which was defined as a baseline uptake value at most of tumor involvement of at least 1.5 times the average standardized uptake value (SUV) of the liver, were eligible [28]. The following exclusion criteria were used: white blood cell count < 2.5 × 10^9^/L, hemoglobin count < 9.0 g/dL, platelet count < 75 × 10^9^/L, serum creatinine > 150 μmol/L, serum albumin > 3.0 g/dL, total bilirubin > 60 μmol/L, cardiac insufficiency, and claustrophobia [28].

### Synthesis of ^68^ Ga-PSMA-617, ^18^F-FDG, and ^177^Lu-EB-PSMA-617

The radiolabeling of ^68^ Ga-PSMA-617 and ^177^Lu-EB-PSMA-617 was conducted as previously described [27]. ^18^F-FDG was synthesized in-house with an 11-MeV cyclotron (CTI RDS 111; Siemens).

### PET/CT acquisition and interpretation

Within 1 week, both ^68^ Ga-PSMA-617 and ^18^F-FDG PET scans were conducted using a dedicated PET/CT scanner (PoleStar m660; SinoUnion Healthcare Inc., Beijing, China). For ^68^ Ga-PSMA-617 PET/CT, the images were acquired at 50–60 min after the administration of ^68^ Ga-PSMA-617 (1.8–2.2 MBq [0.05–0.06 mCi]/kg) [29]. For ^18^F-FDG PET/CT, the patients were instructed to fast for at least 6 h. PET/CT images were obtained at 60–80 min after the intravenous injection of ^18^F-FDG (5.55 MBq [0.15 mCi]/kg). All patients started with a low-dose CT scan (120 keV; 50 mAs) from head to proximal thigh for attenuation correction and anatomical localization, followed by a PET scan at 2 min/bed position. The acquired data were reconstructed using ordered subset expectation maximization (SinoUnion PoleStar: 2 iterations; 10 subsets; Gaussian filter of 4 mm in full width at half maximum; 192 × 192 image size).

The images were transferred to MIM software (Version 7.1.4, MIM Software Inc., Cleveland, USA) and were interpreted independently by two experienced nuclear medicine physicians blinded to the result of another tracer and relevant clinical information. The volume of interest of tumor was segmented using PET Edge, a gradient-based segmentation algorithm [30]. Any focal accumulations of ^68^ Ga-PSMA-617 and ^18^F-FDG that were higher than the surrounding background activity and could not be explained by physiological or benign tracer uptake were interpreted as tumors. The number and SUVmax of tumors were recorded.

### Treatment regimen and follow-up

The ^177^Lu-EB-PSMA-617 radiopharmaceutical was diluted into 100 mL of normal saline and slowly administered intravenously to the patients for 25–30 min. Before that, the patients were infused with normal saline for 30 min for intravenous hydration, and salivary glands were cooled with an ice pack for 30 min to minimize dry mouth syndrome. The patients received up to 3 cycles of PSMA RLT, at 8–10-week intervals.

The clinical data and laboratory profiles, including patients’ subjective health complaints, routine blood examination results, hepatic and renal function indicators, were recorded every 2 weeks. Adverse events were categorized according to the Common Toxicity Criteria for Adverse Events 5.0. The therapeutic effect was evaluated by ^68^ Ga-PSMA-617 and ^18^F-FDG PET/CT at 8 weeks after RLT based on the modified PERCIST 1.0 criteria [31].

### Statistical analysis

All statistical analyses were conducted using SPSS 26.0 software (IBM Corp., Armonk, NY, USA). The quantitative data were presented as the mean ± standard deviation. For data analysis, two-sided Student’s t test was applied to compare the SUVmax of ^68^ Ga-PSMA-617 and ^18^F-FDG PET/CT. Statistical comparison of the tumor numbers was made using Wilcoxon signed-rank test and Friedman’s rank test. The correlation analysis was performed using Spearman correlation coefficient. A *P* value < 0.05 was considered statistically significant.

## Results

### Characteristics of the enrolled patients

We enrolled 30 patients with ACC (15 males and 15 females; average age, 43.0 ± 12.2 years; range, 23–66 years; median, 43 years), including a primary ACC patient, 9 patients with local recurrence, 2 patients with intracranial metastasis, 8 patients with bone metastasis, 5 patients with liver metastasis, 23 patients with lung metastasis, and a patient with axillary lymph node metastasis. The characteristics of the patients are summarized in **Table ****1**. Finally, a total of 4 patients (**no. 4, 9, 10**, and **11)** received ^177^Lu-EB-PSMA-617 treatment with approximately 1.85 GBq (50 mCi). No adverse events were reported or observed in any patient during the radiopharmaceuticals administration.

**Table 1:** Clinical features of 30 ACC patients

No.	Sex	Age	Pathological classification	Involvement of ACC	Stage	Treatment history	Time interval (month)
1	F	53	Cribriform	Maxillary sinus	Primary	None	1
2	F	29	Solid	Larynx	LR	I	99
3	F	54	Cribriform	Nasopharynx	LR	I + II	52
4	M	23	Solid	Bone	LR + DS	I + II + III	8
5	M	47	Solid	Maxillary sinus; liver; bone	LR + DS	I + II + III + IV	19
6	M	34	Cribriform	Maxillary sinus; lung	LR + DS	I + II + III	95
7	M	49	Cribriform	Maxillary sinus; lung	LR + DS	I + II + III	74
8	F	60	Solid	Maxillary sinus; brain; lung; bone	LR + DS	I + II + III	127
9	M	62	Solid	Maxillary sinus; lung; bone; liver	LR + DS	I + II + III + IV	21
10	M	56	Solid	Maxillary sinus; lung; bone; liver	LR + DS	I + II + III	12
11	F	41	Solid	Meninx	DS	I + III	20
12	F	31	Mixed	Lung	DS	I + II + III	61
13	M	32	Cribriform	Lung; bone	DS	I + II + III + IV	75
14	M	32	Mixed	Lung	DS	I + II + III + IV	37
15	M	34	Tubular	Lung; liver; bone; lymph node	DS	I + II + III + IV	124
16	F	28	Cribriform	Lung	DS	I + II + III + IV	127
17	F	39	Mixed	Lung	DS	I + II + III	118
18	M	28	Cribriform	Lung	DS	I + II + III + IV	99
19	M	53	Cribriform	Lung	DS	I + II + III	56
20	M	42	Cribriform	Lung	DS	I + II + III + IV	51
21	M	39	Mixed	Lung	DS	I + II + III + IV	35
22	M	54	Tubular	Lung	DS	I + II + III	84
23	F	30	Cribriform	Lung	DS	I + II + III + IV	154
24	F	45	Solid	Lung	DS	I + II + III	37
25	M	49	Solid	Lung	DS	I + II + III + IV	38
26	F	56	Solid	Bone	DS	I + II + III + IV	109
27	F	25	Cribriform	Lung	DS	I + II + III + IV	25
28	F	44	Cribriform	Lung	DS	I + II + III + IV	51
29	F	66	Mixed	Lung	DS	I + II + IV	60
30	F	54	Cribriform	Lung	DS	I + II + III + IV	60

I surgery; II radiotherapy; III chemotherapy; IV targeted therapy

*ACC* adenoid cystic carcinoma, *Time interval* time interval from diagnosis to PET/CT, *LR* local recurrence, *DS* distant metastases

### Diagnostic performance of ^68^ Ga-PSMA-617 and ^18^F-FDG PET/CT

#### Comparison of tumor detectability

^68^ Ga-PSMA-617 exhibited PET-positive lesions as follows: 1 primary maxillary sinus neoplasm, 9 recurrent tumors, 8 intracranial lesions, 91 bone metastases, 47 liver metastases, 1 lymph node metastasis and 202 lung metastases, for a total of 359 lesions. As a contrast, ^18^F-FDG identified 1 primary tumor, 7 recurrent tumors, 4 intracranial metastases, 86 bone metastases, 42 liver metastases, 1 lymph node metastasis and 301 lung metastases, for a total of 442 lesions. Regarding bone metastases, there were 11 PSMA + /FDG- lesions and 6 PSMA-/FDG + lesions; the combination of two scans can detect 97 bone lesions. For lung metastases, there were 5 foci of PSMA + /FDG- and 104 PSMA-/FDG + , respectively. It is worth noting that CT can exhibit 358 pulmonary nodules, which were interpreted as tumors. The details are shown in Tables 2 and 3.

**Table 2:** Number of PET-positive lesions detected on ^68^ Ga-PSMA-617 and ^18^F-FDG PET/CT

No.	Extrapulmonary metastases						Lung metastases		
	PSMA			FDG			PSMA	FDG	CT
	Primary	Recurrent	Metastases	Primary	Recurrent	Metastases			
1	1	–	–	1	–	–	–	–	–
2	–	1	–	–	1	–	–	–	–
3	–	1	–	–	1	–	–	–	–
4	–	1	4	–	0	3	–	–	–
5	–	1	21	–	1	18	–	–	–
6	–	1	–	–	1	–	15	22	26
7	–	1	–	–	1	–	9	13	19
8	–	1	10	–	1	8	10	10	10
9	–	1	9	–	0	7	7	4	7
10	–	1	36	–	1	33	4	2	4
11	–	–	1	–	–	0	–	–	–
12	–	–	–	–	–	–	12	15	20
13	–	–	30	–	–	30	12	18	25
14	–	–	–	–	–	–	0	9	11
15	–	–	20	–	–	20	10	14	16
16	–	–	–	–	–	–	7	19	22
17	–	–	–	–	–	–	4	11	17
18	–	–	–	–	–	–	10	17	21
19	–	–	–	–	–	–	9	19	24
20	–	–	–	–	–	–	16	16	16
21	–	–	–	–	–	–	14	16	21
22	–	–	–	–	–	–	11	13	13
23	–	–	–	–	–	–	10	11	11
24	–	–	–	–	–	–	9	20	20
25	–	–	–	–	–	–	0	3	3
26	–	–	16	–	–	14	–	–	–
27	–	–	–	–	–	–	12	19	22
28	–	–	–	–	–	–	0	6	6
29	–	–	–	–	–	–	8	11	11
30	–	–	–	–	–	–	13	13	13
Sum	157			141			202	301	358
*P*	0.016*						< 0.001*		

^*^Difference is statistically significant

**Table 3:** Number of PET-positive lesions detected by ^68^ Ga-PSMA-617 PET/CT and ^18^F-FDG PET/CT

ACC lesions	^68^ Ga-PSMA-617 PET/CT alone	^18^F-FDG PET/CT alone	Combination of two modalities	*P*
Extrapulmonary lesions	157	141	163	0.001*
Primary tumor	1	1	1	Not applicable
Local recurrence	9	7	9	0.135
Bone metastases	91	86	97	0.019*
Liver metastases	47	42	47	0.111
Intracranial metastases	8	4	8	Not applicable
Lymph node metastasis	1	1	1	Not applicable
Pulmonary lesions	202	301	306	< 0.001*
Total	359	442	469	< 0.001*

*ACC* Adenoid cystic carcinoma

^*^Difference is statistically significant

In short, ^68^ Ga-PSMA-617 exhibited more PET-positive extrapulmonary tumors (157 *vs.* 141, *P* = 0.016) than ^18^F-FDG. The number of PET-positive pulmonary lesions detected by ^68^ Ga-PSMA-617 was less than ^18^F-FDG (202 *vs.* 301, *P* = 0.001). The combination of ^68^ Ga-PSMA-617 and ^18^F-FDG can detect 469 PET-positive lesions, which was superior to each alone (469 *vs.* 359 *vs.* 442, *P* < 0.001).

#### Comparison of tumor uptake

**Fig. 2:**
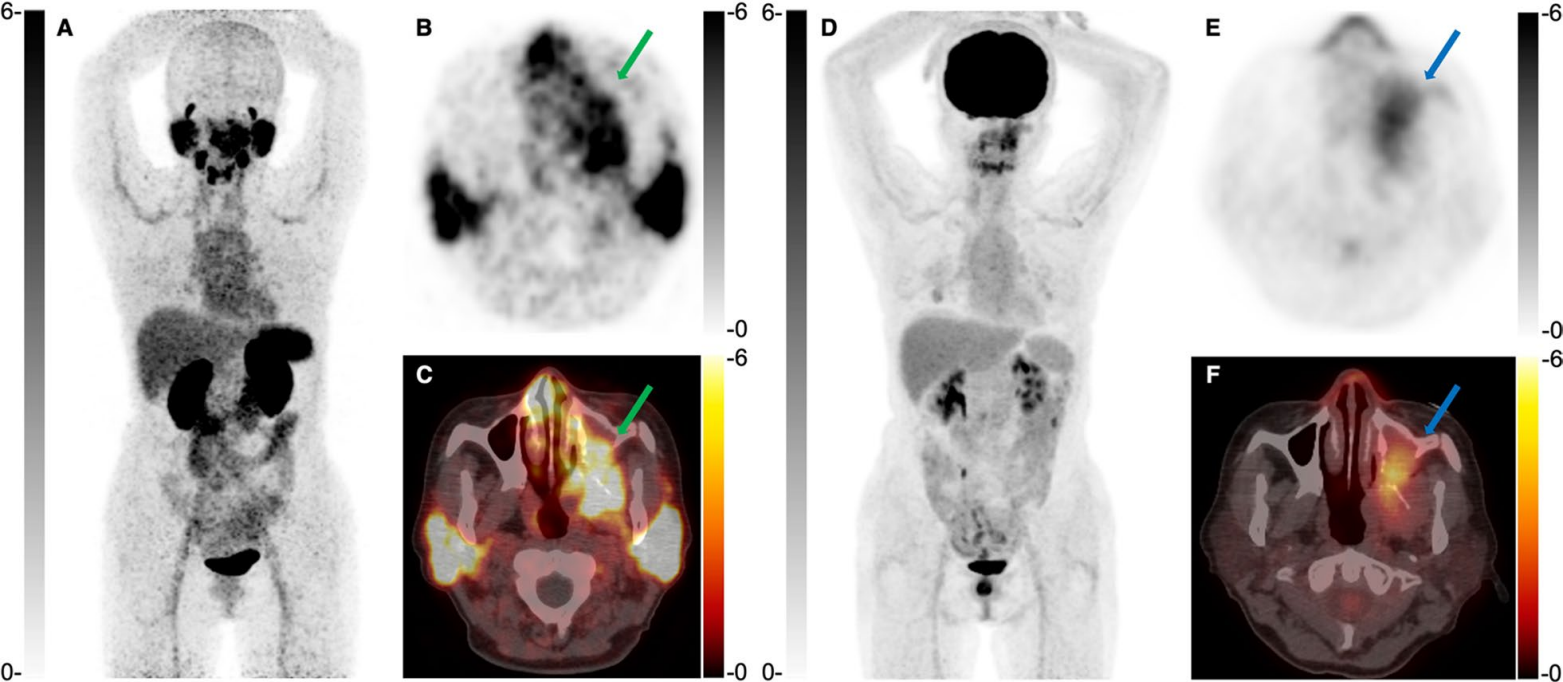
A 53-year-old female patient with primary ACC. Anterior maximum intensity projection (MIP) and axial ^68^ Ga-PSMA PET/CT (**A**–**C**) showed increased tracer uptake of the tumor in the left maxillary sinus (green arrow, SUVmax 9.8). ^18^F-FDG PET/CT (**D**–**F**) showed a lower uptake (blue arrow, SUVmax 6.3)

**Fig. 3:**
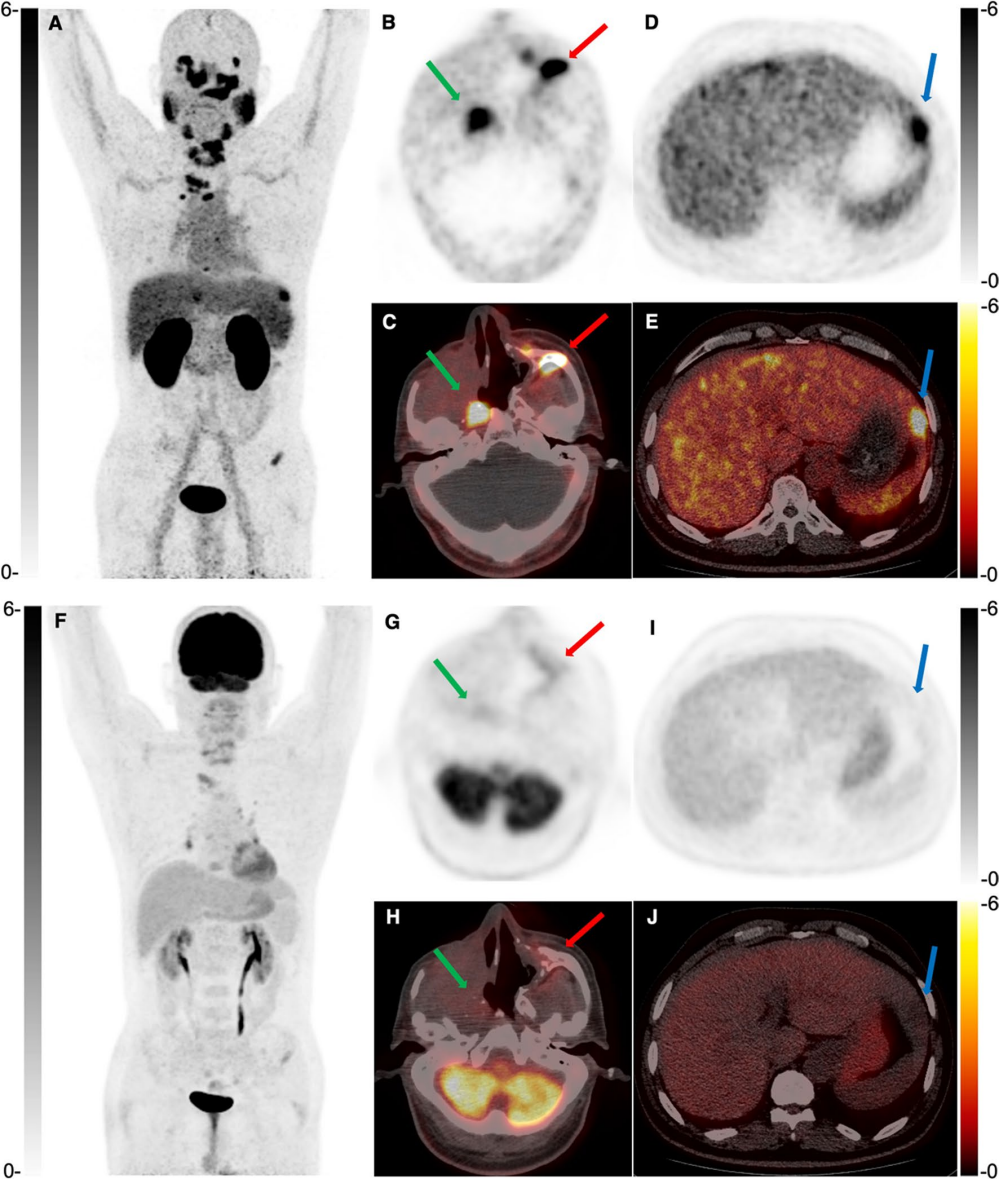
A 62-year-old man was diagnosed with local recurrence and distant metastases 21 months after surgical removal of a right maxillary sinus ACC. ^68^ Ga-PSMA PET/CT (**A**–**E**) revealed a PSMA-avid tumor in the right maxillary sinus (green arrow, SUVmax 11.2), multiple bone metastases (red arrow, SUVmax 16.2), and liver metastases (blue arrow, SUVmax 8.8). ^18^F-FDG PET/CT (**F**–**J**) showed negative recurrent and metastatic foci

**Fig. 4:**
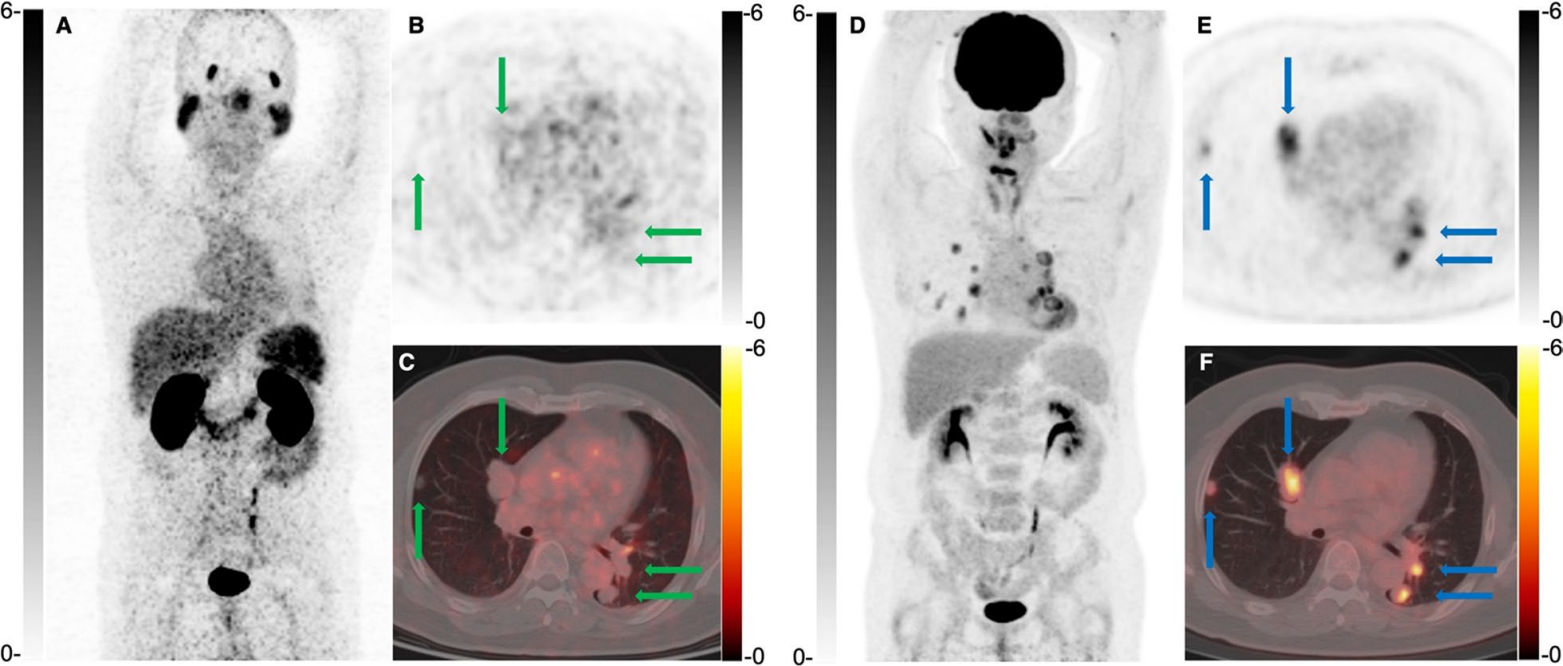
A 49-year-old man was diagnosed with local recurrence and distant metastases 74 months after surgical removal of a left maxillary ACC. ^68^ Ga-PSMA PET/CT (**A–C**) showed multiple lung metastases with negligible uptake (green arrow). ^18^F-FDG PET/CT (**D**–**F**) revealed FDG-avid pulmonary lesions (blue arrow, SUVmax 7.1)

^68^ Ga-PSMA-617 PET/CT exhibited higher tumor uptake than ^18^F-FDG PET/CT in a primary ACC tumor (SUVmax: 9.8 *vs.* 6.3) and 9 recurrent lesions (SUVmax: 10.4 ± 3.8 *vs.* 6.3 ± 5.9, *P* = 0.135), as shown in Figs. 2 and 3. For patients with distant metastases, ^68^ Ga-PSMA-617 PET/CT demonstrated lower tumor SUVmax than ^18^F-FDG PET/CT (4.1 ± 3.6 *vs.* 5.0 ± 3.9, *P* = 0.016), as shown in Fig. 4. Recurrent tumors revealed higher ^68^ Ga-PSMA uptake than metastatic lesions (10.4 ± 3.8 *vs.* 4.1 ± 3.6, P < 0.001), whereas the difference of ^18^F-FDG uptake in recurrent tumors and metastases was not statistically significant (6.3 ± 5.9 *vs.* 5.0 ± 3.9, P = 0.445).

On lesion-based analysis, for extrapulmonary tumors, ^68^ Ga-PSMA-617 PET/CT depicted higher tumor uptakes (8.8 ± 3.6 *vs.* 6.4 ± 4.2, *P* = 0.027) than ^18^F-FDG PET/CT. Regarding pulmonary lesions, ^68^ Ga-PSMA-617 PET/CT illustrated significantly lower SUVmax than ^18^F-FDG PET/CT (3.1 ± 3.0 *vs.* 4.2 ± 3.9, *P* < 0.001).

The SUVmax of tumors, both on ^68^ Ga-PSMA-617 and on ^18^F-FDG PET/CT, was not correlated with patients age, sex, pathological type, history of treatment, or the time interval from diagnosis to PET/CT scan.

### Safety of and therapeutic response to ^177^Lu-EB-PSMA-617 in a patient with ACC

Patient no. 11 accepted three cycles of PSMA RLT, and Patients no. 4, 9, and 10 only accepted one cycle of therapy due to the impact of COVID-19 pandemic.

#### Clinical Symptoms and safety evaluation

The subjective symptoms of pain reported by all 4 patients were improved, with the reduced visual analogue scale (5.0 ± 1.4 for pre-therapy *vs.* 2.8 ± 1.3 for post-therapy, *P* = 0.125).

Patient no. 11 suffered from grade 2 anemia. Patient 10 had been experiencing mild hepatic insufficiency (ALT 75 U/L; AST 68 U/L) and was treated using hepatinica before PSMA RLT. Hence, this patient had no significant liver dysfunction. Routine blood examination, liver and renal function examinations of other 2 patients demonstrated no noticeable fluctuations within therapy. Besides, patients 9, 10, and 11 experienced Grade 1 nausea and fatigue during the observation period.

#### Molecular imaging response

**Fig. 5:**
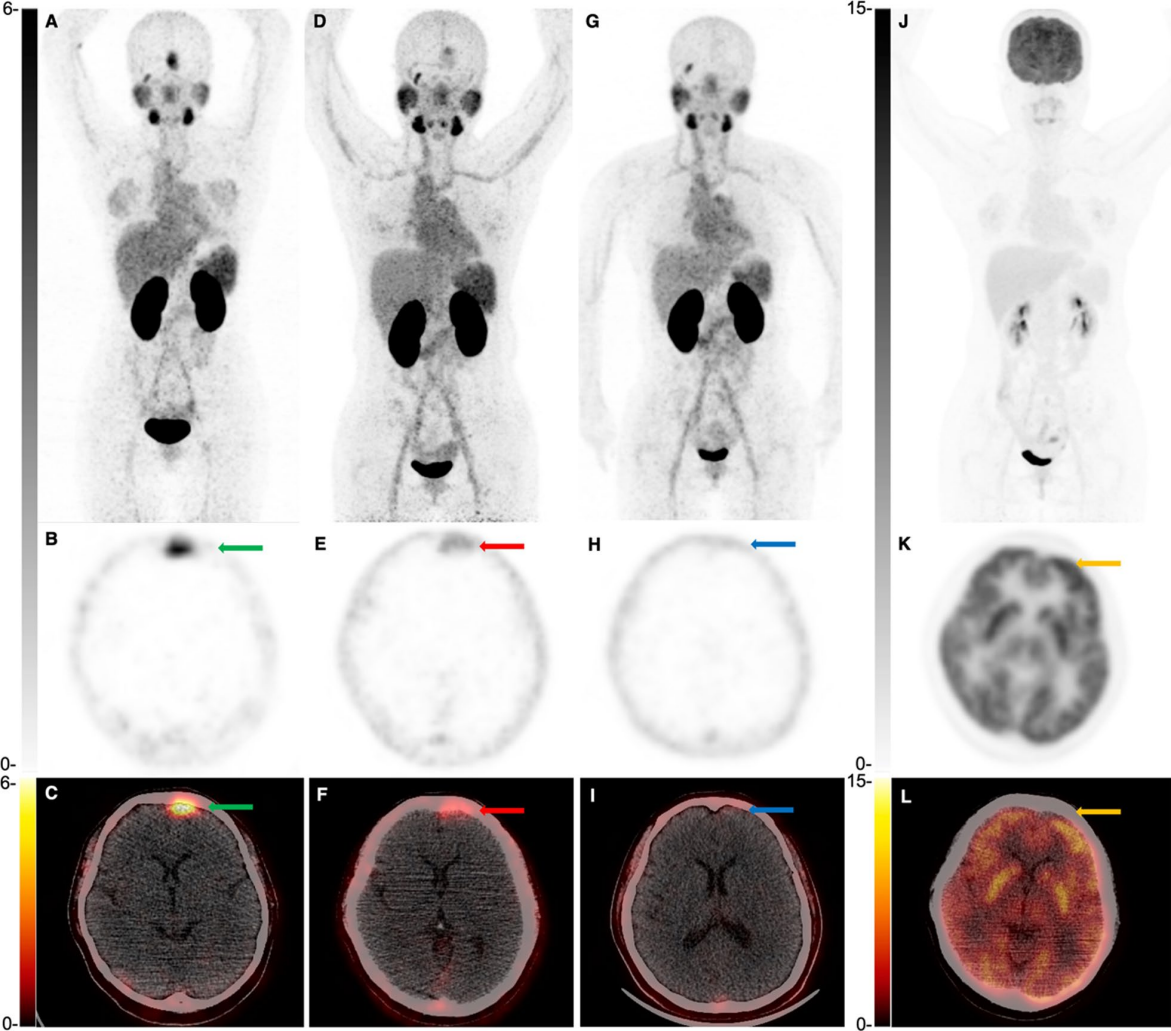
A 41-year-old female patient was diagnosed with a left frontal meningeal metastasis 20 months after surgical removal of the left lacrimal gland ACC. Pre-therapy ^68^ Ga-PSMA-617 PET/CT (**A**–**C**) demonstrated intense PSMA uptake of tumor (green arrow, SUVmax 7.0). ^68^ Ga-PSMA-617 PET/CT reexamination at 9 weeks after the 1st cycle of PSMA RLT (**D**–**F**) and 8 weeks after the 3rd cycle of PSMA RLT (**G**–**I**) revealed significantly decreased tracer uptake of tumor (red arrow, SUVmax 3.5; blue arrow, SUVmax 1.1), which reached the level of CR according to modified PERSIST criteria. However, ^18^F-FDG PET/CT (**J**–**L**) always exhibited no positive lesions (orange arrow)

For PSMA PET response, patient 4 showed encouraging therapeutic effect and the SUVmax of meningeal metastasis decreased from 7.0 to 1.1 (equivalent to the background activity), which achieved CR, as shown in Fig. 5. Patient 11 also demonstrated positive therapeutic response, with reduced tumor uptakes (12.0 ± 3.2 for pre-therapy *vs.* 7.9 ± 3.5 for post-therapy, *P* = 0.031), which reached PR. The therapeutic responses of patients 9 and 10, however, were heterogeneous. Of them, recurrent tumors, lung metastases, and liver metastases showed reduced tumor uptakes (recurrent tumors: 10.9 *vs.* 9.5; lung metastases: 3.4 ± 2.3 *vs.* 1.8 ± 1.5, *P* = 0.036; liver metastases: 8.9 ± 1.3 *vs.* 8.0 ± 1.4, *P* = 0.012). Bone metastases demonstrated increased SUVmax of tumors (9.2 ± 3.3 *vs.* 10.6 ± 2.3, *P* = 0.001).

**Fig. 6:**
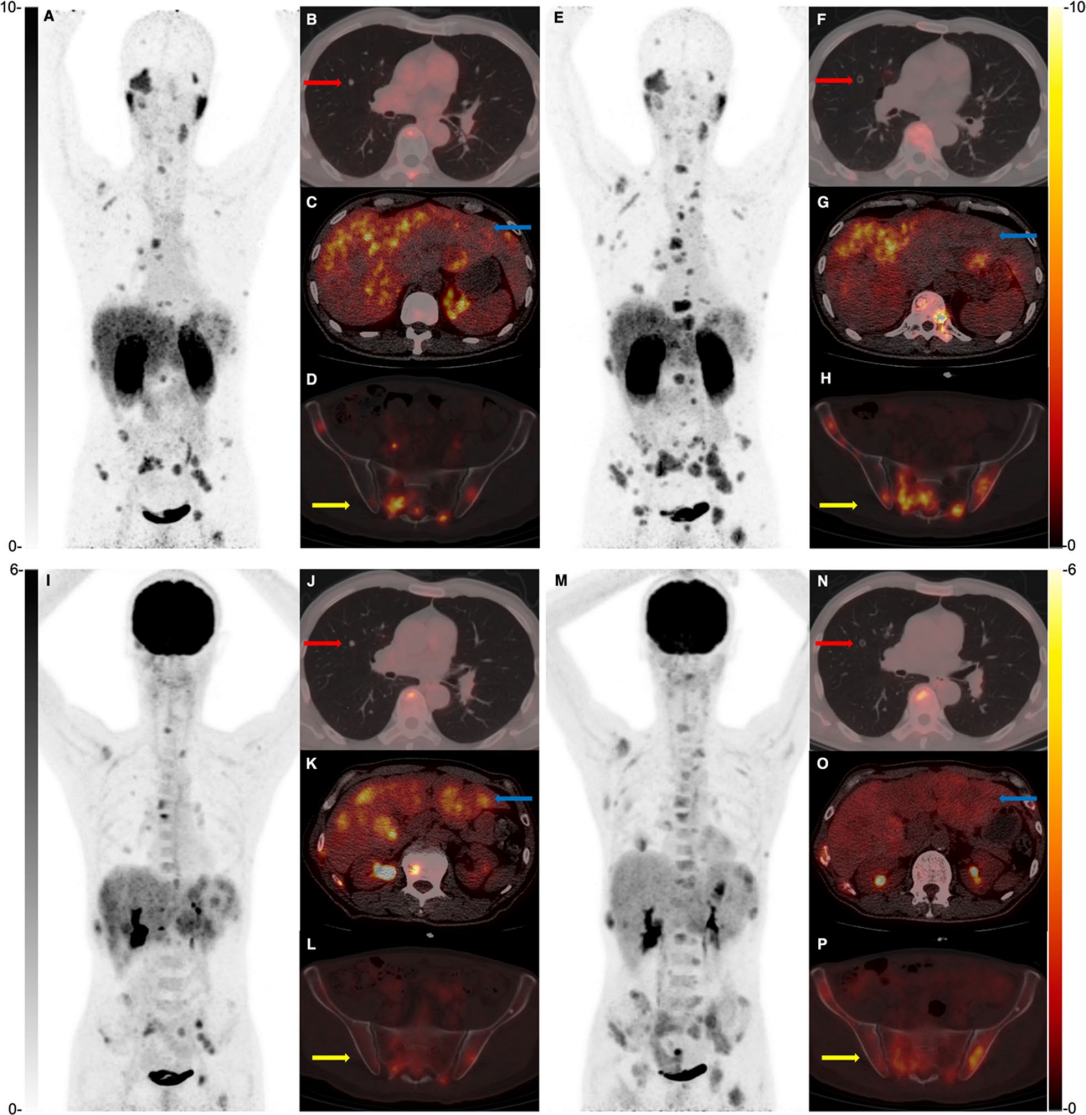
A 56-year-old male patient was diagnosed with local recurrence and multiple metastases 12 months after surgical removal of nasal ACC. Pre-therapy ^68^ Ga-PSMA-617 (**A**–**D**) and ^18^F-FDG PET/CT (**I**–**L**) depicted neoplasm recurrence, multiple liver, bone, and lung metastases. ^68^ Ga-PSMA-617 PET/CT (**E**–**H**) and ^18^F-FDG PET/CT (**M**–**P**) reexamination at 8 weeks after the 1st cycle of PSMA RLT revealed the decreased SUVmax of lung metastases (red arrow) and liver metastases (blue arrow); and CT showed liquefied necrosis occurred inside the lung nodule (red arrow). The uptakes of bone metastases (yellow arrow), however, were significantly increased

For FDG PET response, patient 11 had no FDG-positive lesions. The results of FDG PET response for others were similar to PSMA. Patient 4 depicted reduced uptake of ^18^F-FDG in tumors (2.5 ± 0.6 *vs.* 1.5 ± 0.3, *P* = 0.250). Patients 9 and 10 also exhibited lower SUVmax of tumors after therapy (recurrent tumors: 4.1 *vs.* 3.4; lung metastases: 2.2 ± 0.8 *vs.* 2.0 ± 0.5, *P* = 0.036; liver metastases: 4.7 ± 0.5 *vs.* 1.9 ± 0.2, *P* = 0.002), except for bone metastases (4.0 ± 2.2 *vs.* 5.6 ± 1.9, *P* = 0.006), as shown in Fig. 6.

## Discussion

This is a prospective head-to-head comparison of detection capability between ^68^ Ga-PSMA-617 and ^18^F-FDG PET/CT in the same group of ACC patients and the first clinical study of ^177^Lu-EB-PSMA-617 therapy in ACC.

We found that ^68^ Ga-PSMA-617 PET/CT is superior to ^18^F-FDG PET/CT in detecting extrapulmonary lesions. As previously mentioned, a negative surgical margin plays a decisive role in the prognosis of primary ACC patients, which requires the preoperative diagnosis of the location and extent of tumor to be as accurate as possible. In our study, ^68^ Ga-PSMA-617 PET/CT revealed higher tumor uptake and a larger tumor boundary than ^18^F-FDG PET/CT, which may be a potential advantage over ^18^F-FDG and need to further confirm in a larger sample of patients. For intracranial metastases, it was reasonable that ^68^ Ga-PSMA-617 PET/CT showed better diagnostic performance than ^18^F-FDG PET/CT due to the high physiological accumulation of FDG in the brain. In patients with recurrent tumor, bone metastases, and liver metastases, the diagnostic value of ^68^ Ga-PSMA-617 was also potentially superior to that of ^18^F-FDG. For lung metastases, there was a relatively poor diagnostic effect of ^68^ Ga-PSMA-617, which may be partly attributed to insufficient PSMA uptake in small lung tumor volumes [13, 20]. Besides, we suspect that adenoid cystic carcinoma of the lung contains numerous mucinous secretions within their lumens that may cause relatively low PSMA expression and chronic inflammation of the lungs may also be important reasons. Subsequent studies are needed to confirm the above conjecture [32]. It could be a less significant factor because CT can detect extra pulmonary diseases, which can compensate for the low PSMA PET detection efficiency. We found that the tumor uptake was not correlated with the time interval from diagnosis to PET scan and pathological subtypes, possibly due to the small sample size and heterogeneity of cohort, which will need further confirmation in future studies.

All the above findings are of significance. In fact, for ACC patients after therapy, contrast-enhanced MRI cannot always distinguish between mucosal swelling, inflammatory response, and tumor infiltration [33]. Ruhlmann et al. reported that whole-body FDG PET/CT illustrated high sensitivity in detecting residual/recurrent and regional metastatic spread ACC tumors, which was also superior to that of MRI for local staging and restaging [33]. Furthermore, some studies have revealed that PSMA PET/CT might be useful for detecting lymph node or distant metastases but of limited value for identifying primary tumor or local recurrence [13, 20, 34]. However, in our study, the diagnostic performance of ^68^ Ga-PSMA-617 PET/CT was not inferior to that of ^18^F-FDG PET/CT in patients with primary ACC and local recurrence. The reason for these divergences may be that there have been relatively few head-to-head comparative studies on ACC, and it is impossible to draw generalized, clear conclusions. In our study, ^68^ Ga-PSMA-617 PET/CT combined with ^18^F-FDG PET/CT can achieve better detection efficiency for ACC than each alone and provide more valuable information for the accurate staging, restaging, and treatment of patients.

Another highlight of this study is the exploration of ACC treatment. Because of the lack of effective treatment against ACC, once the patients develop distant metastases, the prognosis is poor. With the successful application of PSMA RLT in PCa [35], this therapy has attracted some attentions in ACC, which also expresses PSMA. Duygu Has Simsek et al. reported that a case with metastatic ACC received PSMA RLT, which achieved a significant pain relief after the administration of 7.5 GBq of ^177^Lu-PSMA [36]. Unfortunately, that patient died in malignancy-induced hypercalcemia without 2nd cycle of ^177^Lu-PSMA therapy. As a new radiopharmaceutical, ^177^Lu-EB-PSMA-617 ensured an excellent therapeutic effect in metastatic castration-resistant prostate cancer [27, 28]. In this study, 2 of 4 patients received satisfactory therapeutic effects, in terms of improvement in both clinical symptoms and imaging response. The other two patients achieved noticeable beneficial results in recurrent foci, liver and lung metastases. But the uptakes of bone lesions were significantly increased, which is unclear whether it is true tumor progression or nonspecific bone uptake (flare phenomenon) [37]. Regrettably, the two patients were not able to continue RLT due to the COVID-19 pandemic. Anyway, all these cases demonstrated that PSMA RLT is a potentially promising treatment for ACC, which would probably benefit more ACC patients.

There are some limitations to our study. The most remarkable issue is the limited number of studied cohorts, especially patients with primary ACC and local recurrence. In addition, only 1 patient underwent 3 cycles of RLT and the others underwent single RLT cycle. Nevertheless, we found obvious clinical significance in the diagnosis and treatment of these patients targeting PSMA. Another limitation is the lack of immunohistochemical PSMA confirmation as a reference standard. Since recurrent and distant metastases are rarely biopsied, it is difficult to obtain tissue samples for immunohistochemistry. However, as previously mentioned, quite a few studies have confirmed the expression of PSMA in ACC. Therefore, our results of PET/CT and PSMA RLT are reliable.

## Conclusion

^68^ Ga-PSMA-617 PET/CT is a valuable imaging modality for the diagnosis and staging of ACC. When combined with ^18^F-FDG PET/CT, they can achieve better diagnostic value for identifying ACC lesions than each alone. PSMA RLT based on ^177^Lu-EB-PSMA-617 may be a promising treatment for ACC. These findings need to be confirmed in further studies with larger cohorts of ACC patients.

## Data Availability

The datasets used and/or analyzed during the current study are available from the corresponding author on reasonable request.

## Abbreviations

ACC: Adenoid cystic carcinoma
PET: Positron emission tomography
CT: Computed tomography
FDG: Fluorodeoxyglucose
PSMA: Prostate-specific membrane antigen
Pca: Prostate cancer
RLT: Radioligand therapy
EB: Evans blue
SUVmax: Maximum standardized uptake value
PERCIST: Positron Emission Tomography Response Criteria in Solid Tumors
